# Novel technique for scapulothoracic arthroscopy: A case report of the application of intraoperative 3D imaging

**DOI:** 10.1016/j.ijscr.2021.106562

**Published:** 2021-11-03

**Authors:** Sy Quyen Nang Vo, Tu Nam Vu, Trung Hieu Pham, Huu Manh Nguyen, Trung Dung Tran

**Affiliations:** VinUniversity, College of Health Sciences, Viet Nam; Vinmec Healthcare System, Center of Sports Medicine and Orthopaedic Surgery, Viet Nam

**Keywords:** Scapuloplasty arthroscopy, Scapulothoracic endoscopy, 3D CT scan, Case report

## Abstract

**Introduction:**

Snapping shoulder syndrome could be effectively treated with scapulothoracic arthroscopy. The excision of the scapular superomedial corner is assumed to help lower the recurrence rate. However, the amount of resection is still controversial. Furthermore, we lack a technique to measure if the resected amount was adequate based only on arthroscopy evaluation.

**Case study presentation:**

We describe a 47-year-old man who suffered from severe snapping shoulder syndrome as a consequence of a deformity of the left superomedial scapular corner. The patient had endoscopic bursectomy and superomedial corner resection. Intraoperative three-dimensional CT scans (3D-CT) were used to evaluate the amount of resection. The patient recovered without incident and resumed his usual activities within 30 days following surgery. At the six-month follow-up, there were no recurrent symptoms.

**Conclusion:**

Intraoperative 3D imaging significantly enhances the safety and efficacy of scapulothoracic arthroscopy. This is a novel technique that, to our knowledge, has not been reported previously in the literature.

## Highlights

• The scapular superomedial corner excision may help reduce recurrence rates of treatment for snapping shoulder syndrome.
• The lack of anatomical landmarks makes estimating the resected volume by arthroscopic vision challenging.
• This is the first report of the application of intra-operative 3D imaging for scapulothoracic arthroscopy.
• Intraoperative 3D imaging significantly enhances the safety and effectiveness of scapulothoracic arthroscopy.
• Intraoperative 3D imaging help in the precise identification of anatomical landmarks in small or complex regions.

## 1 Introduction

Scapulothoracic arthroscopy is performed to treat a variety of conditions, including benign tumors, snapping scapula syndrome, loose-body resection, and bursitis. Snapping scapula syndrome is defined as an audible or palpable clicking of the scapula during movements. Since 1992, scapulothoracic arthroscopy has been used to treat “snapping scapula syndrome” successfully [1]. Nowadays, it is recommended as an effective treatment option for patients who have not responded to non-operative therapy [1], [2], [3]. The treatment includes debridement of the scapulothoracic bursa, with or without excision of the superomedial corner of the scapula. The resection of the superomedial corner may reduce the recurrence rate, especially in severe cases. However, the recommended amount varies among authors and ranges between 1 and 7 cm from the scapular superomedial corner [3], [4], [5] to the lateral margin. If the resection was insufficient, the surgery may be ineffective. On the other hand, excessive resection might cause iatrogenic nerve injury. Some authors determined the amount of resection by measuring the distance from the suprascapular notch [6], while others searched for levator scapulae muscle insertion during the endoscopic process. We illustrate in this article how to employ intraoperative 3D CT scans to assess and correct the resection zone. This case report has been reported in line with the SCARE Criteria [7].

## 2 Case report

**Fig. 1:**
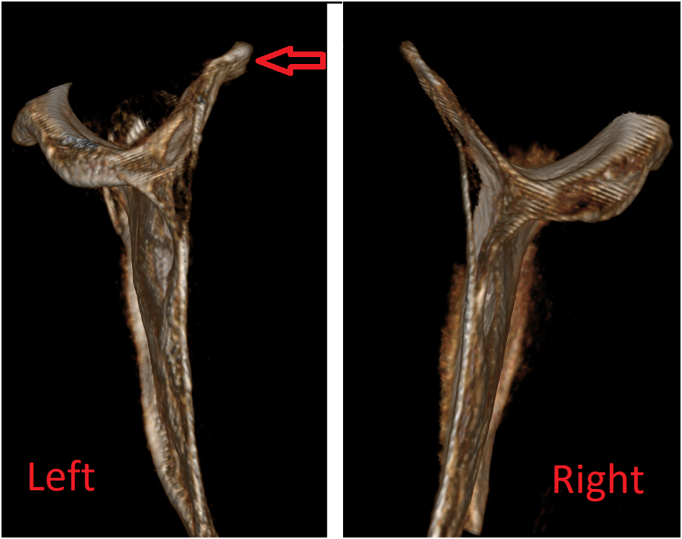
Preoperative CT Scan of Left and Right Scapulas-Note the deformity of the left scapular (Red Arrow)

**Fig. 2:**
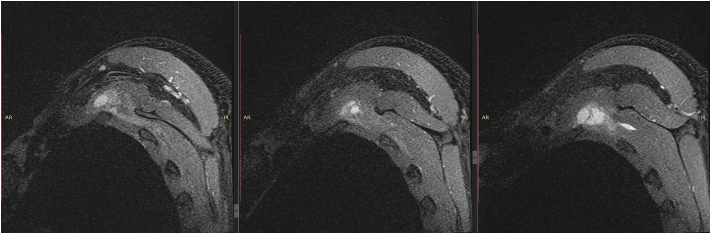
Preoperative MRI revealed bursitis in the left scapulothoracic joint.

A 47-year-old man complained of scapular crepitation whenever he moved his shoulder. He had a motorbike accident 28 months prior that resulted in a claviclasterum dislocation that was treated with open reduction. The sound became increasingly irritating over time, with no improvement despite the use of medications and physical therapy. He was stressed because of the noise and was unable to carry out his daily activities. The physical examination revealed abnormal scapula motion accompanied by snapping sounds during active shoulder movement. CT scans reveal an abnormality in the scapular superomedial corner (Fig. 1) Massive bursitis and thickness of the superomedial scapular corner can also be seen on MRI (Fig. 2).

### 2.1 Surgical techniques

**Fig. 3:**
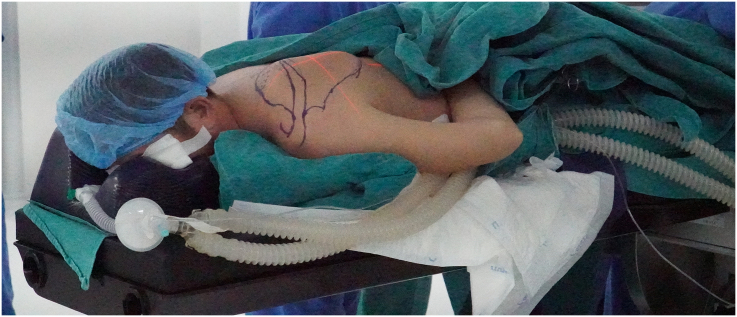
The patient's position.

Under endotracheal anesthesia, the patient was set up in the chicken-wing position [8]. The anesthesia machine and its tubes were put at a position that allowed the robot arm to rotate 360 degrees around the operating table (Fig. 3).

**Fig. 4:**
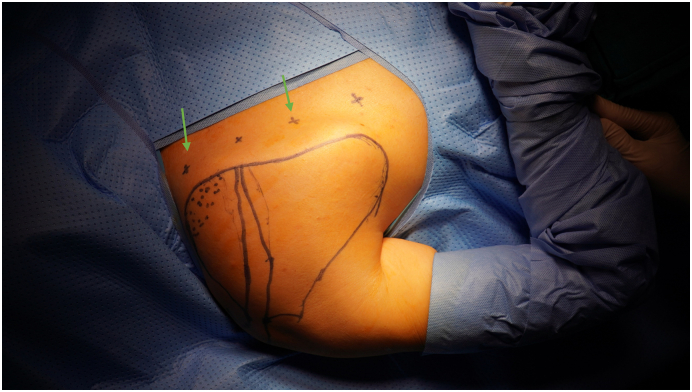
The portrals used (green arrow). (For interpretation of the references to colour in this figure legend, the reader is referred to the web version of this article.)

After calibrating the robot system with the operating table, the patient was covered. The following 2 portals were used in our procedure: (Fig. 4)

• Portal 1: between the scapular spine and the inferior angle of the scapula
• Portal 2: at the level of the superomedial angle (Ejnisman portal) [9], [10]

These portals were located 2 cm medial to the medial margin of the scapula.

**Fig. 5:**
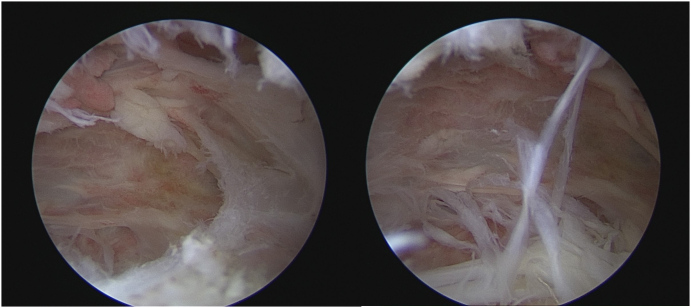
Intraoperative photos-the joint was filled with fibrous tissues.

The arthroscopic probe was inserted through the Ejnisman portal to dissect layers. The serratus anterior muscle served as the roof of this cavity and was also the first anatomical landmark used for orientation. The posterior chest wall, the ribs, and palpable intercostal spaces comprised the floor of this space. We found the serratus anterior space filled with fibrous tissue (Fig. 5).

**Fig. 6:**
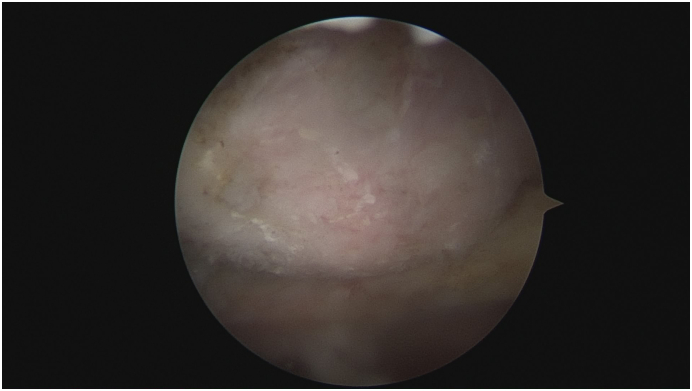
The abnormal bone structure.

After the debridement of inflammatory tissues was carried out, we revealed the abnormal superomedial angle (Fig. 6). Two needles serving as landmarks for the inferior margin and lateral margin of the intended resection zone were inserted under arthroscopic visualization.

**Fig. 7:**
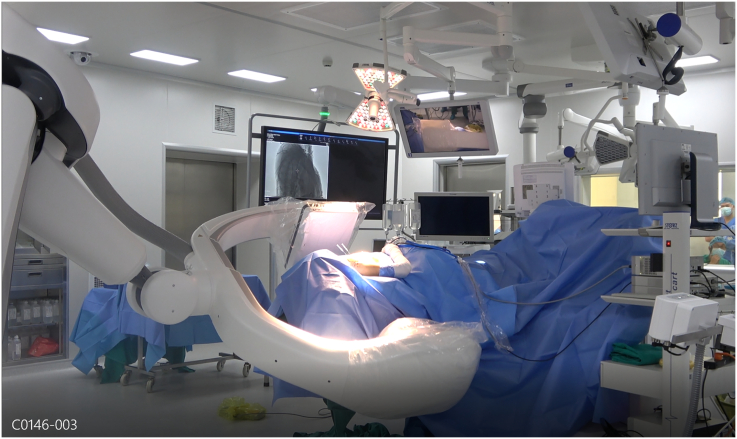
The robot used to perform the 3D CT scan.

**Fig. 8:**
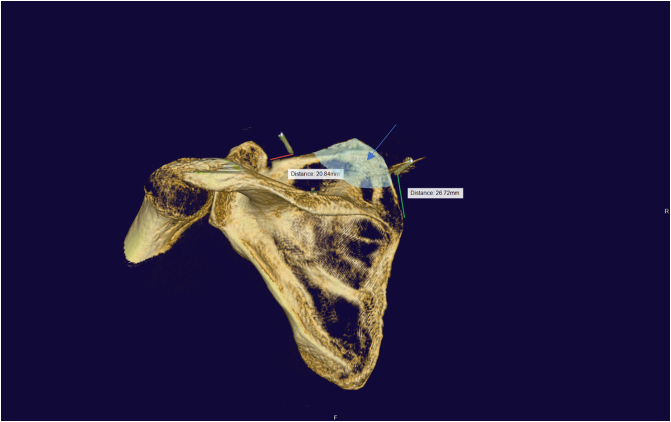
Intraoperative 3D reconstruction image-The top needle was put too near the suprascapular notch and was subsequently repositioned -Blue arrow indicates the intended resection zone. (For interpretation of the references to colour in this figure legend, the reader is referred to the web version of this article.)

A 3D-CT scan of the scapula was performed with Artis Pheno Robot (Siemen) after covering the robot arm (Fig. 7). Another surgeon from outside assisted with the 3D reconstruction of the scapula and checked the position of the needles in relation to the margin of the intended bone area for resection. We aimed to make the distances between the suprascapular notch and the lateral edge of the resection about three centimeters and the distances between the scapular spine and the inferior edge of the resection about two centimeters (Fig. 8). Then, the two marked needles were repositioned to the proper position.

The scapuloplasty procedure was then carried out by using an acromionizer within the level of these needles. It is necessary to thoroughly clean the cavity then, leaving no debris left.

**Fig. 9:**
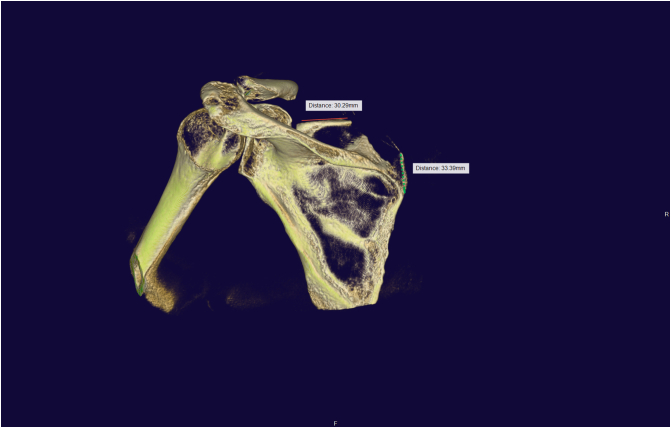
The second intraoperative 3D reconstruction image (after the scapuloplasty).

Following the scapuloplasty, a second 3D CT scan should be conducted to ensure that the proper amount of bone was removed (Fig. 9). Some minor repairs with acromionizer could be required to removed neglected debris.

### 2.2 Post-operation rehabilitation and follow up

**Fig. 10:**
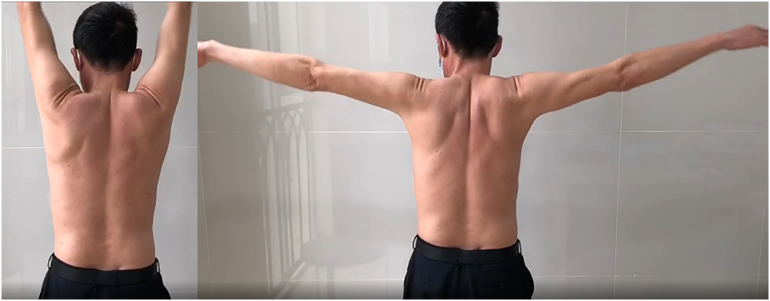
Range of motion at 6-month follow-up.

For comfort, the patient was placed in a sling for two weeks. Maximal protection passive range of motion was performed immediately following surgery. He had a smooth recovery and was back to his normal activities within a month of surgery. After 16 weeks, the patient resumed sports activities. There was no recurring symptoms at the 6-month follow-up (Fig. 10).

## 3 Discussion

Because the superior, inferior, and medial scapula angles are less protected by muscles, even minor anatomical changes at these locations can impair the smooth sliding of the scapula over the ribcage and result in snapping shoulder syndrome. These changes may be asymptomatic until there is an injury (directly to the scapula or indirectly to the superior shoulder suspensory complex) or other causes, for instance: tumor, inflammation, muscle atrophy, nerve palsy [11]. The following anatomical changes in the scapula are frequently reported in the literature: Luschka tubercule, depth of the subscapular fossa, thickness of superior and inferior angles [11], rhinoceros-horn-like projection [12], osteophyte [13].

The superomedial corner, which measured an average of 22.3 mm × 10.8 mm, is an area that was not covered by muscle fibers (between the levator scapulae insertion and the origin of the rhomboid minor) [14]. The supraserratus bursa, which is located here, is one of the most common causes of snapping shoulder syndrome. The surgical treatment option is debridement of the scapulothoracic bursa, with or without excision of the superomedial corner of the scapula. The distance between the suprascapular notch and the lateral edge of the resection varies from 1 to 7 cm depending on different authors [3], [4], [5]. According to Lehtinen et al., an overly curved superomedial angle (>130°) may need a larger resection.

The suprascapular nerve arises from the upper trunk of the brachial plexus to enter the suprascapular notch. This structure could be injured if the bony resection is taken too far laterally. The suprascapular notch is not visualized during the process, and no additional arthroscopic markers are available to anticipate the suprascapular nerve's location. To avoid neurovascular complications, Bell et al. [6] suggested resecting 2 to 3 cm of bone arthroscopically from the superomedial corner of the scapula. They think this is generally sufficient, but the author also stated that in certain patients, the absence of safe anatomical landmarks caused uncertainty regarding how much bone had been removed and how laterally the resection had reached towards the suprascapular nerve [8]. In addition, Bell [8] proposed using a special portal to avoid resecting too much. Lycke [13] recommended establishing an additional superior portal for better visualization if the pathology is located in the superomedial angle. M. Saper [15] advised rotating the working and viewing portals to get a better visualization to achieve the necessary resection.

Some authors utilized 2D conventional fluoroscopy [10] intraoperatively to make scapulothoracic arthroscopy safer, but no author has reported on the use of three-dimensional computed tomography. 3D CT scan is mostly used for preoperative diagnosis [16]. In our case, we make use of the Artis Pheno robot from Siemens for 3D rendering. This is a robotic C-arm that is a part of the Hybrid operating room system. It is mostly utilized in cardiovascular intervention and spine surgery. Another interventional fluoroscopic X-ray device that has a similar function is the IGS Innova (GE Healthcare). Due to the thinness of the scapula and the overlay of the thorax image, it is difficult to detect the border of the scapular superiormedial angle with conventional 2D fluoroscopy. The use of 3D reconstruction not only allows surgeons to view the whole shoulder bone anatomy, but it also allows them to measure the volume of bone that has been removed with remarkable accuracy. With 3D imaging, it is simple to define the distance between the resected zone and anatomical landmarks including the scapular spine and suprascapular notch, which aids in surgical safety. It also helps in the discovery of neglected debris that was not clearly visible in arthroscopic vision.

In our opinion, 3D reconstruction will be particularly useful in some other procedures, for example: removal of benign tumors and loose bodies. The drawback of this method is that it must be performed in a hybrid operating room, which is not widely used in hospitals because of its high cost.

## 4 Conclusion

The use of an intra-operative 3D CT scan improves the safety and efficacy of scapulothoracic arthroscopy. Intraoperative 3D CT has been a relatively new trend in orthopaedic surgery. The strength of this method is that it assists in the accurate identification of anatomical landmarks, particularly in small or complicated areas such as the wrist, shoulder, and pelvis.

## Funding

We declare no funding for this study.

## Ethical approval

The procedures used in this study inhere to the tenets of the Declarations of Helsinki.

## Consent

We introduced the patient to sign informed consent and attached the manuscript.

## Author contribution

- NVSQ contributed to perform the operation
- DTT contributed to revising, and approval for publishing.
- NVT, HPT, MNH contributed to assist the operation, data collection, analysis and interpretation, manuscriptdrafting

## Registration of research studies

This is a case report, so I do not have to register.

## Guarantor

Professor Dung Tran Trung MD, PhD.

## Provenance and peer review

Not commissioned, externally peer-reviewed.

## Declaration of competing interest

We declare that we have no known competing financial interests or personal relationships with anyone that could have appeared to influence the work reported in this paper.
